# Bimodal processing of olfactory information in an amphibian nose: odor responses segregate into a medial and a lateral stream

**DOI:** 10.1007/s00018-012-1226-8

**Published:** 2012-12-27

**Authors:** Sebastian Gliem, Adnan S. Syed, Alfredo Sansone, Eugen Kludt, Evangelia Tantalaki, Thomas Hassenklöver, Sigrun I. Korsching, Ivan Manzini

**Affiliations:** 1 Department of Neurophysiology and Cellular Biophysics, University of Göttingen, Humboldtallee 23, 37073 Göttingen, Germany; 2 Department of Genetics, University of Cologne, Zülpicher Strasse 47a, 50674 Köln, Germany; 3 Cluster of Excellence “Nanoscale Microscopy and Molecular Physiology of the Brain (CNMPB)”, Department of Neurophysiology and Cellular Biophysics, University of Göttingen, Humboldtallee 23, 37073 Göttingen, Germany

**Keywords:** *Xenopus laevis*, Olfactory receptor neurons, *taar* genes, *v1r* genes, *or* class I and class II genes, G proteins

## Abstract

In contrast to the single sensory surface present in teleost fishes, several spatially segregated subsystems with distinct molecular and functional characteristics define the mammalian olfactory system. However, the evolutionary steps of that transition remain unknown. Here we analyzed the olfactory system of an early diverging tetrapod, the amphibian *Xenopus laevis*, and report for the first time the existence of two odor-processing streams, sharply segregated in the main olfactory bulb and partially segregated in the olfactory epithelium of pre-metamorphic larvae. A lateral odor-processing stream is formed by microvillous receptor neurons and is characterized by amino acid responses and Gα_o_/Gα_i_ as probable signal transducers, whereas a medial stream formed by ciliated receptor neurons is characterized by responses to alcohols, aldehydes, and ketones, and Gα_olf_/cAMP as probable signal transducers. To reveal candidates for the olfactory receptors underlying these two streams, the spatial distribution of 12 genes from four olfactory receptor gene families was determined. Several class II and some class I odorant receptors (ORs) mimic the spatial distribution observed for the medial stream, whereas a trace amine-associated receptor closely parallels the spatial pattern of the lateral odor-processing stream. Other olfactory receptors (some class I odorant receptors and vomeronasal type 1 receptors) and odor responses (to bile acids, amines) were not lateralized, the latter not even in the olfactory bulb, suggesting an incomplete segregation. Thus, the olfactory system of *X. laevis* exhibits an intermediate stage of segregation and as such appears well suited to investigate the molecular driving forces behind olfactory regionalization.

## Introduction

The neuronal representation of odors in mammals generally relies heavily on segregation in subsystems that differ anatomically, functionally, and molecularly [1–3]. The two largest subsystems are the main olfactory epithelium (MOE) and the vomeronasal organ (VNO). The MOE contains mostly ciliated neurons expressing the G protein Gα_olf_ and odorant receptors (ORs) that segregate in several distinct expression zones, with class I odorant receptors stringently restricted to one of these zones. The VNO contains two sharply delineated populations of microvillous neurons, a basal layer expressing vomeronasal receptors of type 2 (V2Rs) and Gα_o_, and an apical layer expressing vomeronasal receptors of type 1 (V1Rs) and Gα_i_. The Grüneberg ganglion and the septal organ of Masera constitute two additional olfactory organs [4]. All these sensory surfaces have discrete target areas, the VNO in the accessory olfactory bulb (AOB) and all others within the main olfactory bulb (MOB). Interestingly, lungfish, the closest living relatives of tetrapods, possess several vomeronasal primordia, which share a target region in the olfactory bulb [5, 6]. It is not known whether these primordia are derived from a common ancestral structure in lobe-finned fishes or whether they represent lineage-specific specializations.

In contrast, ray-finned fishes including macrosmatic species [7] possess a single olfactory sensory surface and a common olfactory bulb. Nevertheless, the two main sensory neuron populations, ciliated and microvillous neurons, as well as the main olfactory receptor families and corresponding G proteins all are found in the teleost fish olfactory system intermingled in the shared sensory surface [8–10]. Within the olfactory bulb, the target regions of ciliated and microvillous neurons are somewhat segregated, but are still massively intertwined [11, 12].

*Xenopus laevis*, as an early diverging tetrapod, is evolutionarily much closer to mammals than lungfish, and this also holds true for the structural design of its MOE and VNO. However, *Xenopus* larvae still exhibit a fully aquatic life style. The transition from aquatic to airborne olfaction within the tetrapod lineage likely required a major reconstruction of the olfactory system. The inverse transition necessitated by the secondarily aquatic life style of whales and dolphins did not succeed well, as cetaceans are by and large anosmic species [13]. It is unclear whether the segregation in different subsystems was required by the evolutionary transition to airborne olfaction. Alternatively, the tendency to segregate olfactory functions may long precede this transition. In this case, one would expect evidence of segregation at the molecular and functional level already in the larval olfactory system of *X. laevis*. Some physiological aspects of the larval *Xenopus* olfactory system have already been examined (for a review see [14]). Preliminary information about the larval expression of one vomeronasal and some odorant receptors is available ([15, 16], respectively), and the olfactory receptor gene repertoires of a closely related species, *X. tropicalis*, have been established [17]. However, there has been no attempt so far to correlate molecular analysis and physiological function. Here we examined the spatial distribution of olfactory receptor molecules, associated G proteins, and odor responsiveness in the main sensory surface and the olfactory bulb as well as the nose–brain connections at the same defined stage in *Xenopus* larval development shortly before the onset of metamorphosis. We identified a lateral stream of odor-processing characterized by responses to amino acids, the presence of Gα_o_/Gα_i_ and the expression of trace amine-associated receptors (TAARs), which is segregated from a medial stream of odor processing, characterized by expression of class II ORs, responses to alcohols, aldehydes, and ketones and presence of Gα_olf_. We report that significant spatial segregation of odor processing occurs already in the sensory surface and that segregation of these two odor streams is enhanced in the olfactory bulb.

## Materials and methods

### Tracing of neuronal processes

For visualization of glomerular clusters (see also [18]) in the MOB of larval *X. laevis*, axons of olfactory receptor neurons (ORNs) were labeled using biocytin (ε-biotinoyl-l-lysine, Molecular Probes, Leiden, The Netherlands). All procedures for animal handling and tissue dissections were carried out according to the guidelines of the Göttingen University Committee for Ethics in Animal Experimentation. Briefly, animals (stages 50–54; staged after [19]) were anesthetized with 0.02 % MS-222 (Sigma, Deisenhofen, Germany) for at least 1 min to produce complete immobility. Subsequently, small crystals of biocytin were placed into both nasal cavities, platinum electrodes (0.22 mm in diameter) were inserted into the cavities and square pulses with alternating polarity (30 V, 20 ms, 12 pulses at 1 Hz) were applied to facilitate biocytin intake into ORNs. To allow anterograde axonal transport of the dye, the animals were then kept in water tanks at low light levels for approximately 1 day. The animals were then cooled to produce complete immobility, killed by transection of the brain at its transition to the spinal cord, and fixed in 4 % formaldehyde solution for 2 h at room temperature. A block of tissue containing the olfactory organs, olfactory nerves, and the forebrain was then excised and processed as described below.

To visualize the epithelial location of ORNs that project to the lateral or medial glomerular clusters, ORN axons of the lateral and medial axonal tracts were labeled by electroporation of biocytin. Briefly, a block of tissue (see above) was excised, and all tissue ventral to the olfactory bulb was cut off to get access to the axonal sorting zone. The olfactory nerve and the olfactory organs were left intact. The explant was subsequently transferred to a recording chamber filled with bath solution and a patch pipette (resistance 5–8 MΩ) filled with bath solution saturated with biocytin (Molecular Probes) was carefully inserted into the axonal tract close to its glomerular target region. Lateral and medial axonal tracts of the left and right MOB were, respectively, labeled. Electroporation was performed by application of square pulses (100 V, 20 ms, 12 pulses at 1 Hz). The tissue block was then kept in bath solution for 4 h to allow retrograde axonal transport of biocytin and was subsequently fixed in 4 % formaldehyde solution for 2 h at room temperature.

The formaldehyde-fixed preparations were washed in PBS, embedded in 5 % low-melting point agarose (Sigma) and sectioned at 70 μm on a vibratome (Leica VT 1200S, Bensheim, Germany). The sections were then washed with PBS containing 0.2 % Triton X-100 (PBS-TX) for 15 min and incubated with Alexa Fluor 488 conjugated avidin (100 μg/ml in PBS-TX; MoBiTec, Göttingen, Germany) for 2 h at room temperature. The sections were then washed in PBS for 15 min, transferred to slides, and mounted in mounting medium (Dako, Hamburg, Germany). All tissue sections were viewed using a confocal laser-scanning microscope (LSM 510/Axiovert 100 M, Zeiss, Jena, Germany).

### Preparations of acute slices of the MOE and nose–brain preparations

Larval *X. laevis* (stages 50–54) were killed as described above. For slices of the MOE, a block of tissue containing the olfactory organs, the olfactory nerves, and the forebrain was cut. The tissue was then glued onto the stage of the vibroslicer, cut horizontally into a 150-μm-thick slice, and kept in bath solution. For low magnification imaging of axon terminals in whole olfactory bulbs, tadpoles were anesthetized and ORNs were stained with Fluo-4 10 kDa dextran (Molecular Probes) via electroporation (as described above for biocytin). At least 24 h later, the animals were killed and tissue blocks containing the olfactory systems were excised. The connective tissue covering the ventral side of the telencephalon was removed prior to confocal microscopy. For imaging individual glomeruli, we used nose–brain preparations with the dorsal surface of the olfactory bulbs removed using the vibroslicer. The olfactory organs and the olfactory nerves were left intact. For a more detailed description of these preparations, see earlier work of our lab [20, 21].

### Solutions, staining protocol, and stimulus application

The bath solution consisted of (in mM): 98 NaCl, 2 KCl, 1 CaCl_2_, 2 MgCl_2_, 5 glucose, 5 Na-pyruvate, 10 HEPES, 230 mOsmol/l, pH 7.8. All bath solution chemicals were purchased from Merck (Darmstadt, Germany) or Sigma and were of the highest purity available. Tissue preparations (see above) were transferred to a recording chamber, and bath solution containing 50 μM Fluo-4/AM (Molecular Probes) was added. Fluo-4/AM was dissolved in DMSO (Sigma) and Pluronic F-127 (Molecular Probes). The final concentrations of DMSO and Pluronic F-127 did not exceed 0.5 and 0.1 %, respectively. Cells of the MOE and the olfactory bulb of larval *X. laevis* express multidrug transporters [22, 23] with a wide substrate spectrum, including calcium-indicator dyes. To avoid transporter-mediated destaining of the slices, 50 μM MK571 (Alexis Biochemicals, Grünberg, Germany), an inhibitor of multidrug transporters, was added to the incubation solution. The preparations were incubated on a shaker at room temperature for 35 min. As odorants, we used a mixture of alcohols, aldehydes, and ketones, and mixtures of amines, bile acids (obtained from crude ox bile), and amino acids, all purchased from Sigma (listed in Table 1). Furthermore, we used forskolin (Sigma) as an activator of adenylate cyclase. The odorant mixtures were dissolved in bath solution (stocks of 10–50 mM) and used at a final concentration of 100–200 μM. Forskolin was dissolved in DMSO (stock of 10 mM) and used at a final concentration of 50 μM. In all experiments, the odorant mixtures were repeatedly applied in random order at a minimal interstimulus interval of 2 min. Bath solution was applied by gravity feed from a storage syringe through a funnel drug applicator to the recording chamber. The tip of the applicator was placed directly above the MOE. The odorants and forskolin were applied into the funnel without stopping the flow. Outflow was through a syringe needle placed close to the MOE.

**Table 1:** Components of odorant mixtures

Odorant mixture	Components^a^
Amino acids (AA) (nose–brain prep)	l-Proline, l-valine, l-leucine, l-isoleucine, l-methionine, glycine, l-alanine, l-serine, l-threonine, l-cysteine, l-arginine, l-lysine, l-histidine, l-tryptophan, l-phenylalanine
Amino acids (AA) (MOE acute slices)	l-Proline, l-valine, l-leucine, l-isoleucine, l-methionine, glycine, l-alanine, l-serine, l-threonine, l-cysteine, l-asparagine, l-glutamine, l-arginine, l-lysine, l-histidine, l-glutamate, l-aspartate, l-tryptophane, l-phenylalanine
Bile acids (BA) (main components)	Taurocholic acid, glycocholic acid, cholic acid, deoxycholic acid
Amines (AM)	2-Phenylethylamine, tyramine, butylamine, cyclohexylamine, hexylamine, 3-methylbutylamine, *N*,*N*-dimethylethylamine, 2-methylbutylamine, 1-formylpiperidine, 2-methylpiperidine, *N*-ethylcyclohexylamine, 1-ethylpiperidine, piperidine
Mixture of alcohols, ketones and aldehydes (AL)	α-Terpineol, β-ionone, β-phenylethylalcohol, γ-phenylpropylalcohol, Citral

^a^All components used at a final concentration of 100–200 μM

### Ca^2+^ imaging and data evaluation

Changes of intracellular calcium concentrations of individual ORNs or glomeruli were monitored using a laser-scanning confocal microscope (LSM 510/Axiovert 100 M, Zeiss). Fluorescence images (excitation at 488 nm; emission >505 nm) of the MOE (acute slice of the MOE) or the olfactory bulb (nose–brain preparations) were acquired at 1.27 Hz and 786-ms exposure time per image with ten images taken as control images before the onset of stimulus application. The thickness of the optical slices excluded fluorescence detection from more than one cell layer or glomerulus. Image analysis was performed using custom programs written in MATLAB (MathWorks, Natick, MA, USA). To facilitate selection of regions of interest, a “pixel correlation map” was obtained by calculating the cross-correlation between the fluorescence signals of a pixel to that of its immediate neighbors and by then displaying the resulting value as a grayscale map [24]. The fluorescence changes for individual regions of interest (ORNs or glomeruli) are given as Δ*F*/*F* values. For more detailed information, see our previous work [25]. For whole olfactory bulb imaging, low-resolution Δ*F*/*F* data was processed using a Gaussian filter and is presented as a semi-transparent overlay of the peak response onto the olfactory bulb structure.

In order to quantify the spatial distribution of ORNs responding to amino acids or forskolin, the MOE was subdivided into three parts with equal length of the enclosing borders (see Fig. 3a) using the image-processing and analysis tool ImageJ (http://rsbweb.nih.gov/ij/). Cells with particular response specificities were counted in each of the three MOE subdivisions.

### Immunohistochemistry

The following antibodies were used: Gα_olf/s_ (Santa Cruz Biotech, cat-no. sc-383, lot H1409); Gα_i3_ (Santa Cruz Biotech, cat-no. sc-262, lot E0710, also cross-reacting with other Gα_i_ proteins according to manufacturer information); Gα_o_ (Abcam, Ab35150, lot GR39385-1); Gα_q/11_ (Santa Cruz Biotech, cat-no. sc-46972, lot A0208); Gα_q_ (Santa Cruz Biotech, cat-no. sc-393, lot D0510); anti-tubulin (acetyl K40, Abcam, Ab11323, lot 6-11B-1); Alexa Fluor 488 goat anti-mouse (MoBiTec, cat-no. AZA11001); Alexa Fluor 546 goat anti-mouse (MoBiTec, cat-no. AZA11003); Alexa Fluor 488 goat anti-rabbit (MoBiTec, cat-no. AZA11008); Alexa Fluor 546 goat anti-rabbit (MoBiTec, cat-no. AZA11010); Alexa Fluor 488 rabbit anti-goat (MoBiTec, cat-no. A11078).

Antisera directed against the G protein α-subunits Gα_olf/s_, Gα_o_ and Gα_i_ were used to localize different subsets of receptor neurons (see [15, 26, 27]) in the olfactory organ of larval *X. laevis* and to determine their projection pattern into the olfactory bulb. In some cases, ORNs were visualized by retrograde biocytin labeling (see [28]). Gα_q/11_ and Gα_q_ only stained non-sensory supporting cells (data not shown).

Larval *X. laevis* (stages 50–54) were killed as described above. Tissue blocks (see above) were fixed in 4 % formaldehyde solution for 2 h at room temperature, equilibrated in 30 % saccharose, and embedded in Jung tissue freezing medium (Leica) for cryosectioning (10–20 μm sections). Sections were washed in PBS-TX, and non-specific binding was blocked with 3 % normal goat serum (ICN, Aurora, OH, USA) for Gα_i_ stainings, or 3 % bovine serum albumin (Sigma) for Gα_olf/s_ and Gα_q/11_ stainings, or 2 % bovine serum albumin for Gα_o_ and Gα_q_, in PBS-TX for 1 h. The sections were then incubated overnight at 4 °C with the primary antibodies (1:200) diluted in the respective blocking solution/PBS. Primary antibodies were washed off with PBS and the respective fluorophore-coupled secondary antibodies were applied at a dilution of 1:500 in 1 % blocking solution/PBS for 2 h at room temperature. The secondary antibodies were then washed off in several changes of PBS. To allocate signaling molecule expression to either ciliated or microvillous ORNs, we performed double-labeling experiments with antibodies against Gα_olf/s_, Gα_i_ and Gα_o_ together with antibodies against tubulin (ciliary marker; 1:2,000) or fluorophore-coupled phalloidin (marker for microvilli; 1:250; Alexa Fluor 488 coupled to phalloidin, MoBiTec, cat-no. AZA12379; Alexa Fluor 546 coupled to phalloidin, MoBiTec, cat-no. AZA22283). All preparations were then transferred to slides, mounted in mounting medium (Dako) and viewed using a confocal laser-scanning microscope (LSM 780/upright Axio Examiner Z1, Zeiss). Optical sections were processed using ZEN software (Zeiss) and displayed as maximum intensity projections.

In order to quantify the spatial distribution of G protein-like immunoreactivity, we subdivided the MOE as described in the section above, and summed up the G protein-related fluorescence intensity values of the entire area of each subdivision of the MOE.

### Western-blot analysis

Western blots were performed to test the specificity of antibodies directed against mammalian G alpha protein subunits Gα_olf/s_, Gα_o_ and Gα_i_ in larval *X. laevis*. Tissue from the olfactory organs and the olfactory bulb from larval *X. laevis* (stages 43–45, 52–54 and 64–66) was collected and immediately conserved in liquid nitrogen. The frozen tissue samples were then homogenized using a glass homogenizer in lysis buffer containing 10 mM Tris/HCl, pH 7.4, 0.1 % SDS, 1 mM EDTA pH 8.0, 1 % Triton X100, and a protease inhibitor mixture (Sigma). The lysate was centrifuged at 10,000 × *g* for 1 min, and the protein content in the supernatant was quantified by BCA assay (Thermo Scientific, Rockford, USA). Equal amounts of protein (30 μg per lane) were separated in 10 % SDS-PAGE gels under reducing conditions and then transferred to Amersham Hybond-ECL nitrocellulose membranes (GE Healthcare, Little Chalfont, England) for Western-blot analysis. The membranes were blocked with 5 % non-fat dry milk in PBS-Tween 20 (137 mM NaCl, 2.7 mM KCl, 8 mM Na_2_HPO_4_, 1.46 mM KH_2_PO_4_, 0.05 % Tween 20, pH 7.4) and incubated overnight at 4 °C with 1:1,000 dilution of the primary antibodies (anti-Gα_olf/s_, anti-Gα_o_ and anti-Gα_i_, for details see above). Blots were washed three times with PBS Tween 20 and probed with horseradish peroxidase (HRP)-conjugated secondary antibodies (Dianova, anti-rabbit) for 1 h at a concentration of 1:2,000 at room temperature. The blots were washed again three times with PBS Tween 20, developed using AceGlow *chemiluminescence substrate* (peqlab, Erlangen, Germany) and analyzed by Bio1D software (Vilber Lourmat, Eberhardzell, Germany). The experiments revealed bands in the range of the appropriate molecular weight.

### In situ hybridization

*Xenopus laevis* genomic DNA was extracted using standard protocols and used for PCR-mediated cloning. For this study, 12 genes from four olfactory receptor families were used. Primers were designed using published sequence information for the OR class l (*xb242, xr116, or52d1*) and OR class ll *(xb180, xb177, xgen147*) receptor genes, ([16, 29]; Mezler and Breer, GenBank entries). Primers were designed for two *v1r* genes (*v1r10, v1r11*) reported in [27], and one *v1r* gene (*v1r6*) was cloned using the homology approach with *v1r6* in *X. tropicalis*. The *X. laevis* *v1r6* turned out to share 92 % amino acid sequence identity with its ortholog in *X. tropicalis*. For two TAAR (*taar1, taar4a*) and one *v2r* gene (*xv2r E*-*1*), we used the primer set described in [30] and [15], respectively. The primer sequences we used were: *xr116* (5′-GTGACTCTCCTCTGCTACTT-3′, 5′-AGTAAAAACCGTCCGTCTTG-3′), *xb242* (5′-ACCAATGCAGTGGTATTAGTG-3′, 5′-TGGGTACTAGATTTGTGCTCG-3′), *or52d1* (5′-GAYTCYTTCATCMTYATGCTGATG-3′, 5′-CHAWTARRTGRGTGGTACAGGT-3′), *xb180* (5′-AATGAAGGAGCCACAATGTAC-3′, 5′-GCAATAATGAGTACGCCAATG-3′), *xb177* (5′-TTACCTTCTGATAATCTGGGAG-3′, 5′-AGCAACAATGGACAATACAAC-3′), *xgen147* (5′-CAGTRATGTCCTWTGACAG-3′, 5′-TCCCGGTATTGGACACTATC-3′), *v1r11* (5′-AGYCAACCTCATACTCTACC-3′, 5′-TCTGTCTGTGCTCCTTTTGC-3′), *v1r10* (5′-CAGTTTGCTCAGCTGTTATCAG-3′, 5′-GTSAGATAGTCCRTGTCACAG-3′), *v1r6* (5′-TCATTCTCAATGCCCGTACA-3′, 5′-CCAAAACCATTAGCCCAACA-3′), *taar1* (5′-GCCTTCACAATGGTATTTCTGG-3′, 5′-CCTATCTCTGCTTCGGGACAC-3′), *taar4a* (ACTTGGTCTGTTTCCTGTGTGTTTT-3′, 5′-TGGAAACTATGGTGGTTATGTACAAG-3′), *xv2r E*-*1* (5′-TGAGCTTCCTCCTCCTTGTC-3′, 5′-GGTAATGTCCGAGCTAAAAATGC-3′).

Resulting fragment lengths varied from 200 to 500 bp. All the genes were cloned into pDrive (Qiagen, Hilden, Germany) and later confirmed by sequencing. Antisense probes for in situ hybridization were derived from the cloned DNA by PCR, using the same primer sequences, but one of them with a T3 (TATTAACCCTCACTAAAGGGAA) promoter site attached to the 5′-end. Digoxigenin (DIG) was incorporated into the probes according to the DIG RNA labeling kit supplier protocol (Roche Molecular Biochemicals, Mannheim, Germany). Olfactory organ tissue blocks were prepared as described for immunohistochemistry. Cryostat sections of 10–12 μm were obtained and postfixed in 4 % paraformaldehyde for 10–15 min at room temperature.

Hybridizations were performed overnight at 60 °C using standard protocols. Anti-DIG primary antibodies coupled to alkaline phosphatase (Roche Molecular Biochemicals) and NBT-BCIP (Roche Molecular Biochemicals) were used for signal detection.

In order to quantify the spatial distribution of olfactory receptor genes, the MOE was subdivided into three parts as described above. Areas were nearly identical for the subregion close to the VNO (medial region) and the intermediate region (36 and 35 %, respectively), but somewhat less for the lateral region, most distant from the VNO (29 %). Half of the in situ experiments were randomized and cell count was done blindly with respect to the genes involved. No difference in the results from non-randomized evaluation was seen.

## Results

### The lateral and medial glomerular cluster exhibit distinctly different odor-response profiles

We analyzed the medial-to-lateral spatial distribution of olfactory bulb responses to four main odor groups of aquatic animals, amino acids (feeding stimulants, [31, 32]), amines (also food signals, [32, 33]), bile acids (social interactions, [31, 32, 34]) and alcohols, aldehydes, and ketones [35]. In addition, we analyzed olfactory bulb responses upon mucosal application of forskolin, an activator of the cAMP-dependent transduction pathway of ORNs.

**Fig. 1:**
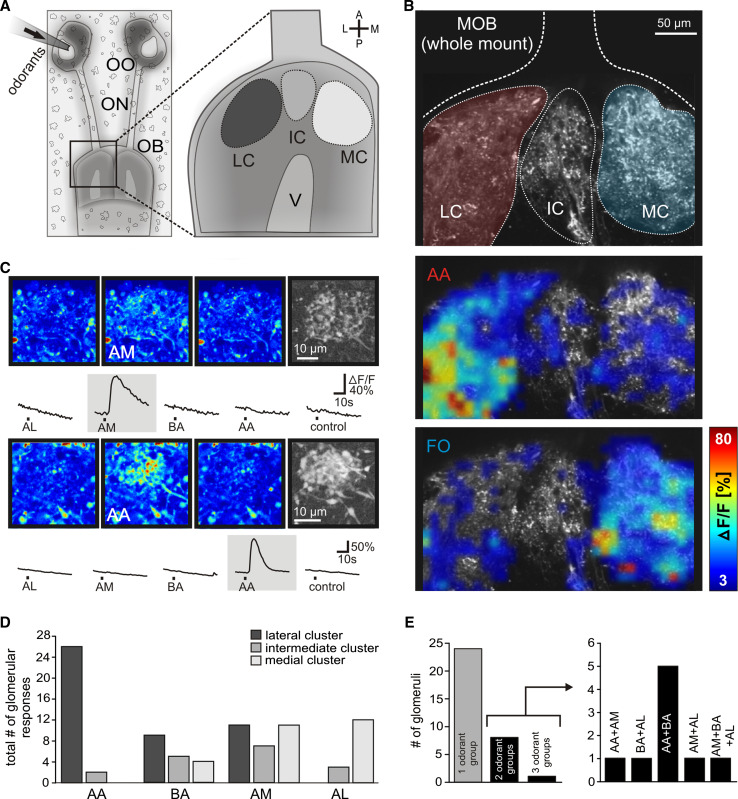
Odorant responses in the glomerular layer of the main olfactory bulb. **a** Schematical representation of the nose–brain preparation (*left panel*) and the three main glomerular clusters of the MOB (*right panel*). **b** Whole-mount olfactory bulb preparation stained with Fluo-4 dextran showing the three main clusters of the MOB (*upper panel*; LC *red*, MC *blue*). Application of amino acids preferentially induced an increase in Ca^2+^-dependent fluorescence in the lateral cluster (*intermediate panel*), whereas forskolin elicited activity predominantly in the medial cluster (*lower panel*). A representative example of seven separate experiments is shown. **c** Sequence of three pseudocolored images showing calcium transients of an individual glomerulus situated in the medial cluster upon application of amines. The images were taken before stimulus application, at the peak of the response and after return to the baseline fluorescence (from *left* to *right*). The fine glomerular structure of the activated glomerulus was visualized by a grayscale correlation map (*rightmost image*; see “Materials and methods” for details). The time courses of the [Ca^2+^]_*i*_ transients of the glomerulus, evoked by mucosal application of the different odorant groups are given below the images. The lower group of pictures shows an individual glomerulus, situated in the lateral cluster, responsive solely to amino acids (same explanation as above). **d** Histogram showing the location of odorant-responsive glomeruli (*n* = 80 glomeruli from 32 nose–brain preparations). **e** Out of 33 glomeruli tested for their responsiveness to all four odorant groups, 24 responded to one odorant group, eight responded to two odorant groups, and one glomerulus to three odorant groups (*left panel*). Odorant profiles of multiresponsive glomeruli are shown in the *right panel*. All odorants were applied at a final concentration of 200 μM, forskolin was applied at a final concentration of 50 μM. *A* anterior, *P* posterior, *L* lateral, *M* medial, *OO* olfactory organ, *ON* olfactory nerve, *OB* olfactory bulb, *AA* amino acids, *AL* alcohols, ketones, and aldehydes, *AM* amines, *BA* bile acids, *FO* forskolin, *LC* lateral glomerular cluster, *IC* intermediate glomerular cluster, *MC* medial glomerular cluster, *V* lateral ventricle, *control* application of bath solution

In low magnification views of the whole olfactory bulb (Fig. 1a), application of amino acids and forskolin to the intact olfactory organ induced transient increases of Ca^2+^-dependent fluorescence of Fluo-4 in the neuropil of the glomerular layer (Fig. 1b). Amino acids preferentially induced responses in the lateral cluster, whereas forskolin elicited activity predominantly in the medial cluster. For analysis of individual glomeruli, we used nose–brain preparations with the surface of the olfactory bulbs removed. Odor-responsive structures were first allocated to one of the known glomerular clusters of the MOB (see Fig. 1a). We then focused on the responding neuropil and repeated the odorant application to verify that the responding spots were individual, clearly delineated glomeruli with the typical fine structure (Fig. 1c, pseudocolored and grayscale images). The reproducibility of glomerular responses was verified in nearly all cases by repeating the odorant application at least twice. The signals were odor-specific, since application of bath solution alone never evoked a comparable response (see Fig. 1c).

As representative examples, we show the response characteristics of two glomeruli to all four odor groups (Fig. 1c). The first glomerulus, a component of the medial cluster, reacts only to amines, the second glomerulus, situated in the lateral cluster, only to amino acids. In total, we analyzed odor responses of 80 glomeruli that could be clearly allocated to a glomerular cluster (*n* = 32, nose–brain preparations; 42, 15, and 23 glomeruli for lateral, intermediate, and medial cluster, respectively; Fig. 1d). Thirty-three glomeruli were tested with all four odor groups, and of these, the large majority reacted only to a single of the four odor groups (Fig. 1e). Of the 24 monoresponsive glomeruli, seven reacted to the mixture of amino acids, six to bile acids, seven to amines, and four to the mixture of alcohols, aldehydes, and ketones. With one exception, the odor responses of the remaining nine glomeruli were restricted to two out of four odor groups. Amine responses were found to co-exist with all three other odor groups, likewise for bile acids, however we never found a glomerulus that reacted to both amino acids and alcohols, aldehydes, and ketones (Fig. 1e).

Clear positional preferences were seen already in the overview of the whole olfactory bulb for amino acid and forskolin responses: amino acid responses were nearly exclusively found in the lateral cluster, while forskolin responses showed a complementary restriction to the medial cluster (Fig. 1b). This result was confirmed by the examination of single glomeruli at higher magnification, where we found an almost exclusive location of amino acid-responsive glomeruli in the lateral cluster (Fig. 1d), consistent with previous observations [18, 36]. Moreover, the glomeruli responsive to alcohols, aldehydes, and ketones were almost exclusively located in the other large cluster, the medial glomerular cluster (Fig. 1d). In contrast, bile acid- and amine-responsive glomeruli were more broadly distributed, and were found in all three cluster regions, lateral, intermediate and medial (Fig. 1d).

### Lateral and medial glomerular cluster in the olfactory bulb are innervated by spatially segregated populations of receptor neurons

**Fig. 2:**
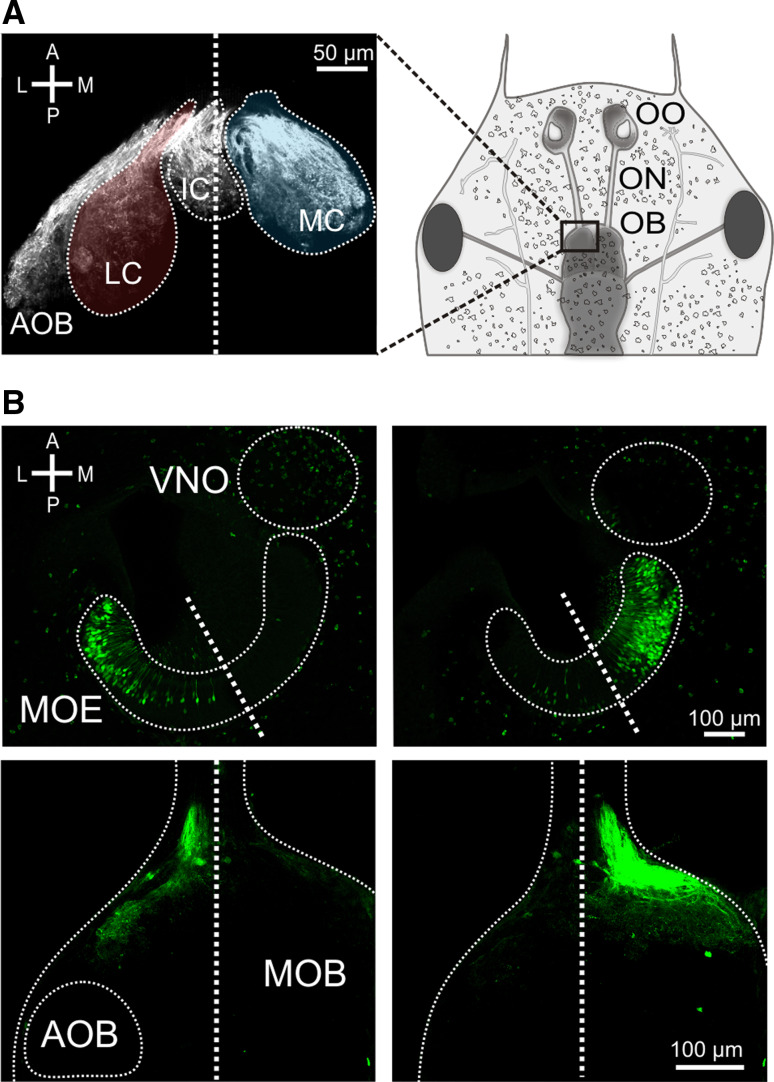
Visualization of spatially segregated streams within the main olfactory system. **a** Glomerular clusters are visualized by anterograde transport of biocytin (*left panel*). The three main glomerular cluster (LC *red*, MC *blue*) as well as the AOB are distinctly visible. The schematic drawing (*right panel*) shows the location of the *left panel* in larval *Xenopus laevis*. **b** Retrograde labeling of ORNs by biocytin electroporation into the lateral (*lower left-hand panel*) and medial (*lower right-hand panel*) axonal tracts at the level of the MOB. *Thick dotted lines* indicate midlines and *thin dotted lines* trace organ outlines. Electroporation into the lateral axonal tract predominantly labeled lateral ORNs (*upper left-hand panel*), whereas electroporation into the medial axonal tract predominantly labeled medial ORNs (*upper right-hand panel*). *A* anterior, *P* posterior, *L* lateral, *M* medial, *OO* olfactory organ, *ON* olfactory nerve, *OB* olfactory bulb, *LC* lateral glomerular cluster, *IC* intermediate glomerular cluster, *MC* medial glomerular cluster

To what extent is the functional segregation apparent in the glomerular response patterns in the olfactory bulb already present in the sensory surface? To analyze this question we used retrograde tracing of biocytin from the axonal tracts emerging from the lateral and medial cluster (Fig. 2a) at very lateral and very medial positions, respectively. For lateral injections, the large majority of biocytin-labeled somata were situated in the lateral part of the MOE, but occasionally cells were also found in the medial region (Fig. 2b). For medial injections, the distribution of labeled somata was the inverse, i.e., the vast majority were positioned in the medial part of the MOE and only rarely were cells found in the lateral region (Fig. 2b). These findings show that the segregation apparent in the olfactory bulb is already present in the olfactory epithelium, some overlap of the two subsystems notwithstanding. These two spatially segregated ORN projection paths will be named lateral and medial stream throughout the manuscript.

The existence of these two subsystems suggests that the receptor neuron sub-populations projecting to the lateral and the medial cluster of glomeruli should have distinct response properties, reflecting that of the respective glomerular clusters. Thus we proceeded to measure calcium responses of individual ORNs in situ using acute slices of the MOE stained with the calcium-indicator dye Fluo-4.

### Most receptor neurons are specific for one of the four odor groups

**Fig. 3:**
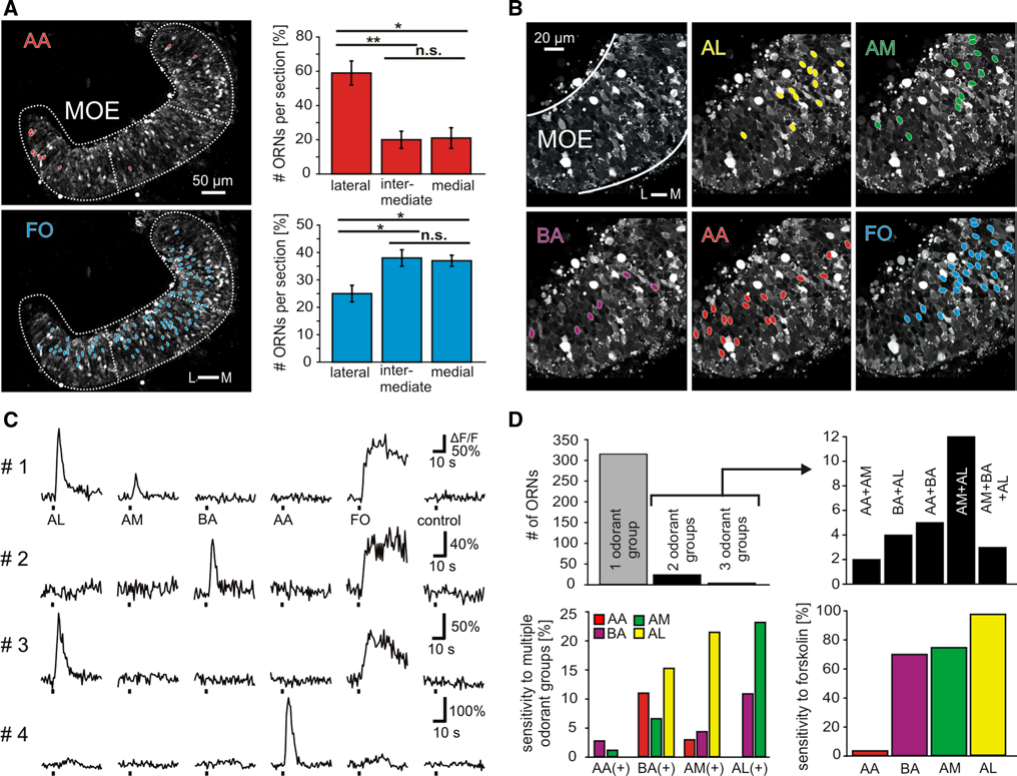
Odorant responses at the level of the main olfactory epithelium. **a** Acute slice preparation of the whole MOE stained with Fluo-4 (image acquired at rest). The *red ovals* indicate somata of individual ORNs that responded to the mixture of amino acids (100 μM). Quantitative evaluation (*right panel*) shows more amino acid-responsive cells in the lateral third of the MOE compared to intermediate and medial segments (*n* = 116 ORNs from 13 acute slices of the MOE). The *blue ovals* indicate somata of forskolin-responsive cells (50 μM) of the same slice preparation. Significantly more forskolin-responsive cells were located in the medial and intermediate third of the MOE compared to its lateral third (*n* = 600 ORNs from 14 acute slices of the olfactory epithelium). Statistical analysis was performed using a *t* test; *p < 0.05, **p < 0.01; *error bars* show SEM. **b** Acute slice preparation of the MOE stained with Fluo-4 (image acquired at rest). Field of view does not cover the whole MOE. The *colored ovals* indicate somata of individual ORNs that responded to the mixture of alcohols, aldehydes, and ketones (*yellow*), amines (*green*), bile acids (*magenta*), amino acids (*red*) or to forskolin (*blue*). All odorants were applied at a final concentration of 200 μM, forskolin at a final concentration of 50 μM. **c** Time courses of [Ca^2+^]_*i*_ transients of four responsive ORNs of this slice. **d** Out of 340 ORNs tested (*n* = 17 acute slices), 314 responded to only one odorant group whereas 23 responded to two odorant groups and three to three odorant groups (*upper left panel*). The exact odorant profile of multiresponsive cells is shown in the *upper right panel*. The *histogram* in the *lower left-hand panel* gives the frequencies of correlated responses to the tested odorant groups. The *lower right-hand panel* gives the correlation of odorant- and forskolin-sensitivity of individual ORNs. *L* lateral, *M* medial, *AA* amino acids, *AL* alcohols, ketones, and aldehydes, *AM* mixture of amines, *BA* mixture of bile acids, *FO* forskolin, *control* application of bath solution

We detected sparse populations of responsive receptor neurons for all four odor groups tested for glomerular responses (Fig. 3a, b). For amino acids and amines, this corroborates earlier observations [28, 30], whereas responses to bile acids and the mixture of alcohols, aldehydes, and ketones had not been examined so far. Representative traces obtained for individual neurons (Fig. 3c) show fast calcium transients for all odor groups, typical for ORN responses (see [37]). As a positive control, we applied the adenylate cyclase activator forskolin, which increases cAMP levels, i.e., activates the cAMP-dependent ciliated receptor neurons (Fig. 3a–d). Application of bath solution by itself never evoked any comparable response (Fig. 3c). The reproducibility of ORN responses was verified by regularly repeating the application of most of the odorants at least twice. In total, we analyzed the odorant responses of 340 ORNs out of 17 MOE slices (Fig. 3b–d).

Amino acid responses were observed much more frequently than those to each of the other three odor classes, with 188 versus 46, 70, and 65 responsive cells for amino acids, bile acids, amines, and the mixture of alcohols, aldehydes, and ketones, respectively. Over 90 % of ORNs responded only to one odor group (Fig. 3d), showing that the vast majority is narrowly tuned to individual groups of odorants, similar to the high tuning specificity described above for individual glomeruli (Fig. 1e). The remaining cells mostly responded to two of the four odor groups and only three cells responded to three odor groups. An example of a mixed odor response is shown in Fig. 3c (top row), whereas the other three cells depicted in Fig. 3c only react to a single odor group each. Amine responses were found to co-exist with all three other odor groups, likewise for bile acids, however we never found a cell that reacted to both amino acids and the mixture of alcohols, aldehydes, and ketones (Fig. 3d). This parallels exactly our observations for glomeruli (see Fig. 1), consistent with a clearly segregated pathway for amino acids versus alcohols, aldehydes, and ketones.

Amino acid-responsive cells were notably more specific than the other three groups, as <5 % of amino acid-responsive cells, but about one-third of amine, bile acid-responsive cells, and cells responsive to the mixture of alcohols, aldehydes, and ketones reacted to other odor groups (Fig. 3d, bottom left panel). We therefore went ahead to measure the spatial distribution of amino acid-responsive neurons.

### Amino acids activate lateral ORNs, while odors signaling via cAMP preferentially elicit responses in medial ORNs

In previous work, we have shown that the MOE of larval *X. laevis* comprises two large subsets of ORNs with differing transduction cascades and a differential sensitivity to amino acid odorants [21, 38]. Here, we have analyzed the spatial distribution of such cells. From the tracing results described above, we expected the tripartite organization of the olfactory bulb in three major glomerular clusters to be somewhat reflected in the sensory surface. We have therefore quantified the number of odor-responsive cells in the lateral, intermediate, and medial third of the olfactory epithelium separately. We find that amino-acid responsive cells were strongly enriched in the lateral third of the olfactory epithelium (Fig. 3a), with about 60 % of all amino acid-responsive cells restricted to the lateral third of the MOE, and the remaining 40 % evenly distributed between intermediate and medial region. This lateral preference corresponds to the spatial pattern for amino acid responsive glomeruli in the olfactory bulb and thus provides functional evidence for the existence of at least two segregated subsystems suggested by our tracing experiments reported above. Interestingly, the lateral-to-medial gradient is steeper in the olfactory bulb, where not a single amino acid-responsive glomerulus was observed in the medial cluster (Fig. 1d), pointing to further sorting-out within the olfactory nerve connecting MOE and olfactory bulb.

Cells responding to the mixture of alcohols, aldehydes, and ketones appeared to exhibit a preferentially medial location, while responses to bile acids as well as amines seemed more evenly distributed through all regions of the olfactory epithelium (data not shown). However, due to low cell numbers, quantification was not reliably possible in these cases. We have therefore chosen to analyze the spatial distribution of cells responding to forskolin, as a summary measure for neurons transducing odor signals via cAMP. From our olfactory bulb responses (see Fig. 1) and from comparison with other species, these may be expected to include three of the four odor groups tested here, amines, bile acids, and alcohols, aldehydes, ketones [12, 39, 40]. Amino acids are expected to mostly signal via a cAMP-independent mechanism [12, 41]; but see [11].

We found forskolin-responsive cells to be moderately depleted in the lateral segment, with no difference between intermediate and medial segments (Fig. 3a). Thus, the forskolin response was inversely distributed to the amino acid response. In terms of absolute numbers of cells counted, the lateral depletion of forskolin is very similar to the lateral enrichment of amino acid-responsive cells, consistent with the notion that these two pathways are strictly segregated and may amount to most or all of olfactory signaling in the MOE.

The analysis of traces originating from individual cells confirms this prediction of exclusivity between amino acid response and forskolin response (Fig. 3c, d). Virtually all amino acid-sensitive ORNs did not respond to forskolin and virtually all ORNs responsive to the mixture of alcohols, aldehydes, and ketones were sensitive to forskolin (Fig. 3d, bottom right panel). Interestingly, while the large majority of bile acid- and amine-responsive cells were activated by forskolin, about one-fourth were not, suggesting that bile acid- as well as amine-responsive cell groups are not homogeneous, but contain cells signaling via cAMP as well as via a cAMP-independent pathway. This heterogeneity of bile acid- and amine-responsive receptor neurons may explain their broader spatial distribution and less narrow chemical tuning compared to the two other groups.

### The lateral sensory surface contains microvillous neurons that express Gα_i_ and Gα_o_, whereas the medial region contains ciliated neurons that express Gα_olf/s_

**Fig. 4:**
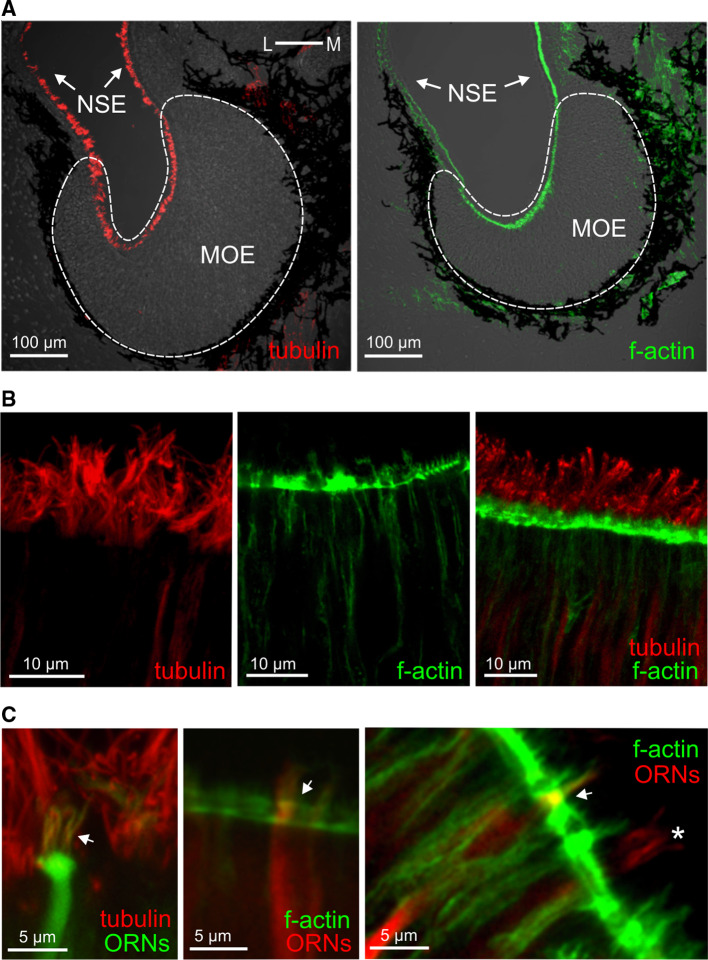
Tubulin and actin identify cilia and microvilli, respectively. **a** Antibodies against tubulin and a marker of f-actin (phalloidin) both labeled structures in the whole MOE and in the adjacent non-sensory epithelium (NSE; tubulin, *left-hand panel*; f-actin, *right-hand panel*). **b** Higher magnifications of the apical MOE show cilia labeled with antibodies against tubulin (*left-hand panel*), microvilli labeled with phalloidin (*middle panel*), and a double-labeled MOE (*right-hand panel*). **c** ORNs and their processes were visualized by nerve backfills with biocytin and double labeled with antibodies against tubulin (*left panel*, ciliated neuron, the* arrow* points to cilia), and phalloidin (*middle and right panels*, microvillous neurons,* arrows* point to olfactory knobs). Another backfilled neuron with somewhat longer processes (*asterisk*) was not labeled by phalloidin, i.e., it is a ciliated neuron

**Fig. 5:**
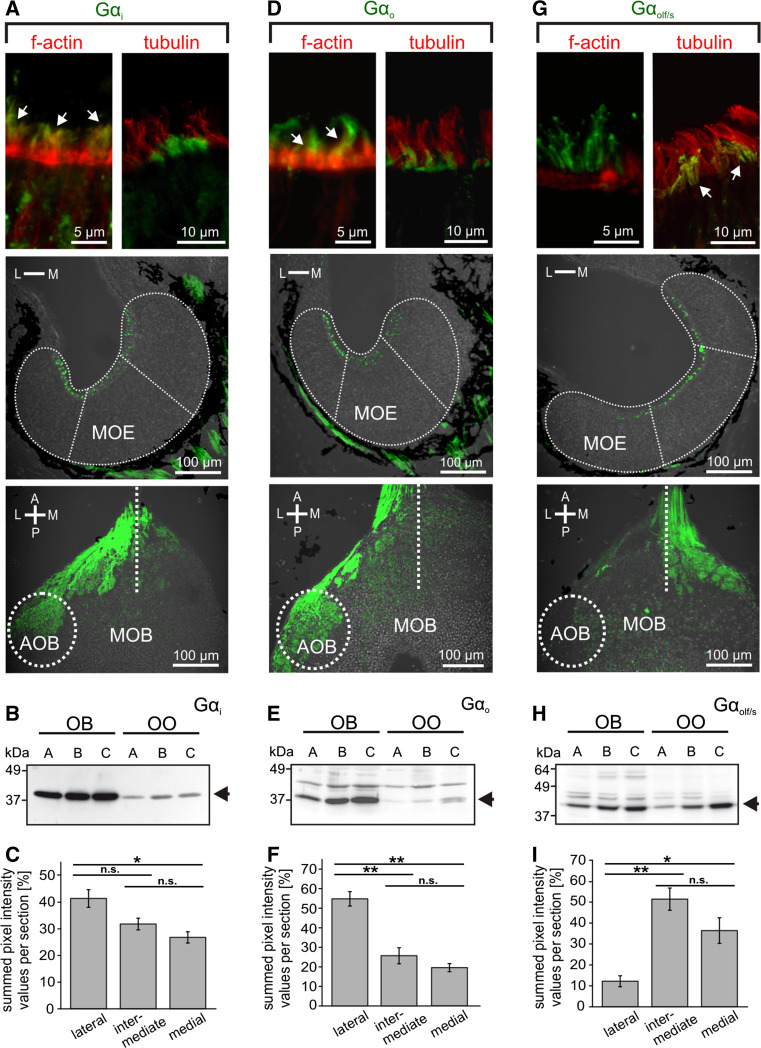
G-protein immunohistochemistry in the main olfactory epithelium and the main olfactory bulb. Antibodies against Gα_i_ (**a**) and Gα_o_ (**d**) preferentially labeled apical structures of ORNs located in the lateral and intermediate part of the MOE (*middle row*) and axon bundles of the olfactory nerve and glomeruli of the lateral and intermediate glomerular clusters of the MOB, as well as glomeruli in the AOB (*lower row*). *Dotted lines* indicate the approximate borders and subdivisions of MOE and olfactory bulb. Gα_i_ and Gα_o_ immunoreactivity was localized in apical endings of phalloidin-positive (*arrows*) and tubulin-negative microvillous olfactory receptor neurons (*upper row*). Gα_olf/s_ (**g**) immunoreactivity showed a complementary distribution, preferentially localized in ORNs and glomeruli of the medial and intermediate regions of MOE and MOB (*middle* and *lower row*, respectively). Gα_olf/s_ in apical endings of ORNs co-localized with the ciliary marker tubulin, but not with f-actin (*upper row*, *arrows*). Western-blot analysis of Gα_i_ (**b**), Gα_o_ (**e**) and Gα_olf/s_ (**h**) antibodies using tissue samples of olfactory organ and olfactory bulb of larval *Xenopus laevis* (**a** stages 43–45, **b** 52–54, and **c** 64–66, respectively). *Arrows* indicate bands corresponding to the predicted molecular weights of Gα_i_ and Gα_o_ (~40 kDa) and Gα_olf/s_ (~44 kDa). The Gα_i_ antibody is highly specific, whereas Gα_o_ and Gα_olf/s_ antibodies show minor crossreactivity to other proteins. Quantification of fluorescence intensity of G protein labeling for Gα_i_ (**c** *n* = 9 MOEs), Gαo (**f** *n* = 7 MOEs) and Gα_olf/s_ (**i** *n* = 5 MOEs). Gα_i_ and Gαo are enriched laterally, whereas Gα_olf/s_ shows clear depletion in the lateral segment. Significance was evaluated by *t* test (*p < 0.05, **p < 0.01; *error bars* show SEM). *A* anterior, *P* posterior, *L* lateral, *M* medial, *OO* olfactory organ, *OB* olfactory bulb

In the mammalian olfactory system, two main signal transduction pathways are known. One is the so-called canonical pathway, which uses Gα_olf_ and cAMP, and is found in ciliated receptor neurons; the other uses Gα_i_ or Gα_o_ and is found in microvillous neurons [2]. Our results with forskolin, detailed above, suggest that the medial subsystem, which is responsive to alcohols, aldehydes, and ketones, signals via cAMP and therefore might be in ciliated receptor neurons, whereas the amino acid-responsive lateral subsystem may be expected to reside in microvillous receptor neurons. To test these hypotheses, we have analyzed the spatial distribution of G proteins both in the olfactory epithelium and the olfactory bulb, alone and in combination with markers for cilia and microvilli (Figs. 4, 5). The latter was necessary, because in larval *X. laevis* the processes of microvillous and ciliated receptor neurons are not much different in length and can therefore not be distinguished unequivocally by morphology (data not shown; see also [42]).

For immunohistochemical localization, we employed antibodies against Gα_i_, Gα_o_ and Gα_olf/s_, whose specificity was confirmed in Western blots of olfactory bulb and olfactory organ (Fig. 5). All three antibodies stain the apical surface, i.e., the apical endings of ORNs, as well as axons in the olfactory nerve and olfactory bulb (Fig. 5a, d and g), but not the cell somata.

Gα_i_-like and Gα_o_-like immunoreactivity showed a graded distribution, decreasing from the lateral to medial area of the MOE (Fig. 5a, c, d, f). A drastically different distribution was observed for Gα_olf/s_-like immunoreactivity, which was strongly enriched in the medial and intermediate part of the MOE, but very minor in the lateral part (Fig. 5g, i). The distributions in the olfactory bulb are even more sharply delineated, with Gα_i_ and Gα_o_ nearly exclusively restricted to the lateral glomerular cluster (and the AOB), and Gα_olf/s_ restricted to glomeruli of the medial and intermediate glomerular cluster (Fig. 5a, d, g; bottom panels). A similar segregation is already seen in the olfactory nerve layer of the olfactory bulb, with the Gα_i_ and Gα_o_ containing fibers preferentially located in the lateral part and the Gα_olf/s_ containing fibers preferentially located in the medial part (Fig. 5a, d, g; bottom panels). Thus, considerable sorting out occurs en route to the olfactory bulb, consistent with the sorting out seen for amino acid-responsive and forskolin-responsive neurons (Figs. 1,  2,  3). These data suggest that the lateral stream signals via Gα_i_ and Gα_o_ and the medial stream may signal via Gα_olf/s_.

To establish the cell types expressing the respective G proteins, we used phalloidin, a marker for f-actin that is abundant in microvilli [43] and antibodies against tubulin as a ciliary marker [44]. Both markers label the whole sensory as well as non-sensory epithelium (Fig. 4), due to the presence of microvilli and cilia also in supporting and non-sensory cells [42]. Double-labeling experiments with G protein antibodies revealed that both Gα_i_-like and Gα_o_-like immunoreactivity are restricted to the apical endings of microvillous ORNs (Fig. 5a, d, upper panel), whereas Gα_olf/s_-labeled structures could be identified as cilia of ORNs (Fig. 5g, upper panel).

Our data suggest the medial olfactory stream to be composed of ciliated receptor neurons and the lateral stream to consist of microvillous receptor neurons.

Microvillous and ciliated receptor neuron populations are expected to express different receptor families. As a first step in linking such molecular features to the two streams, we cloned 12 genes from the four olfactory receptor gene families of *X. laevis* and investigated their spatial distribution within the olfactory epithelium by in situ hybridization.

### Sparse expression patterns of *or* class I, *or* class II, *v1r* and *taar* genes in the larval MOE

**Fig. 6:**
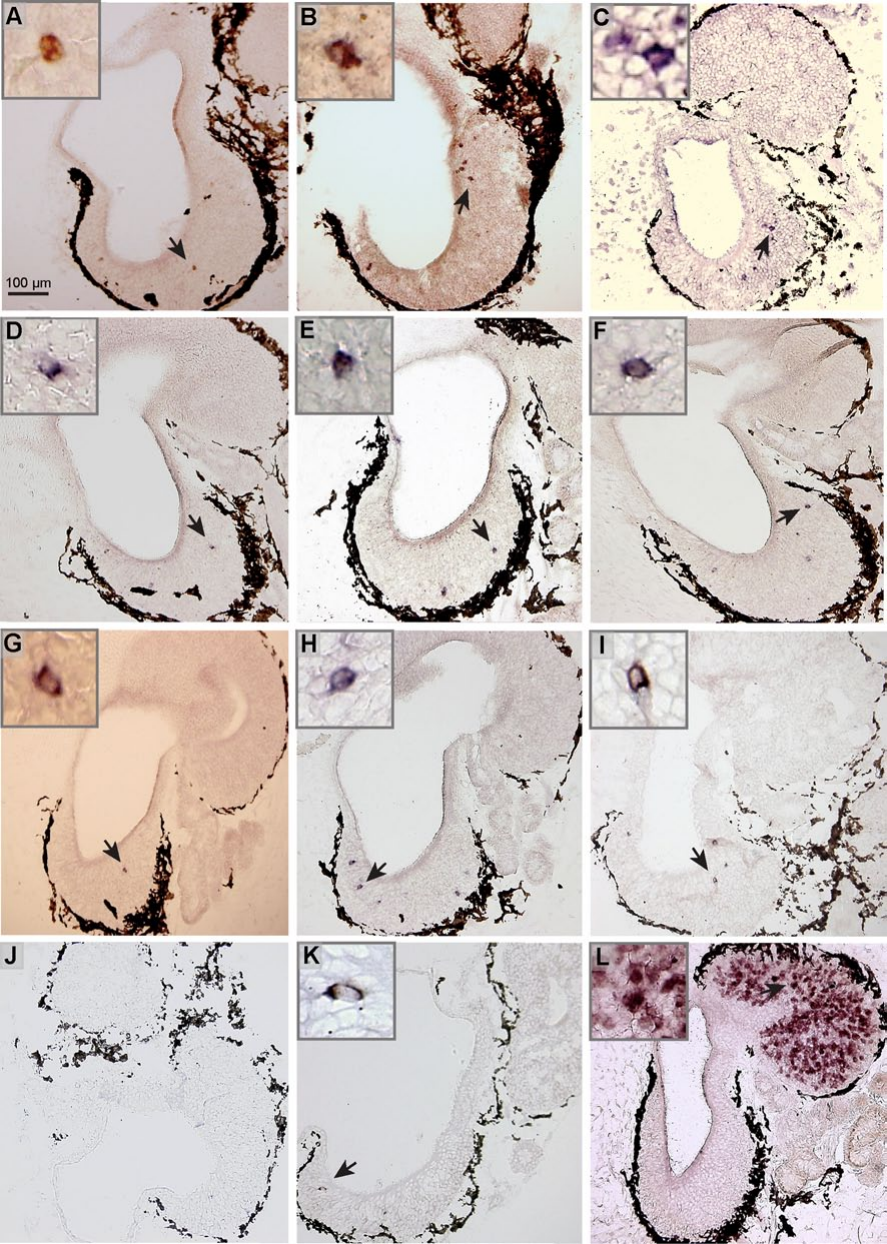
Spatial expression patterns of olfactory receptor genes from the different families. Twelve genes from four olfactory receptor families were cloned by PCR using either the published *Xenopus laevis* sequence information for the primer or degenerated primer based on the *Xenopus tropicalis* sequence. The clones were confirmed by sequencing and riboprobes were prepared. In situ hybridization (**a**–**l**) was performed under stringent conditions, using cryostat sections of larval *Xenopus* nose tissue, which encompassed both the MOE and the VNO. *Insets* show enlargements of cells marked by *arrow*. Class I *or* genes (**a** *xr116*, **b** *xb242*, **c** *or52d1*); class II *or* genes (**d** *xb180*, **e** xb177, **f** *xgen147*); *v1r* genes (**g** *v1r10*, **h** *v1r11*, **i** *v1r6*); *taar* genes (**j** *taar1*, **k** *taar4a*); **l** *v2r* gene *xv2r* *E-1*. Results for *xr116* and *xgen147* are consistent with in situ hybridization results obtained by Mezler and coworkers [16] for similar larval stages, and the result for *xv2r E-1* is consistent with reports by Hagino-Yamagishi and coworkers [15]

We performed in situ hybridizations with members of *or* class I and II, *v1r*, *v2r*, and *taar* olfactory receptor gene families, using RNA probes for a total of 12 different genes. We show that *taar1* was not expressed in the sensory surface (Fig. 6j), and thus may have a non-olfactory role in *Xenopus*, as has been reported for mammalian and fish orthologs of *taar1* [45, 46].

The *v2r* probe was chosen for its extensive crossreactivity with many closely related *v2r* genes. The probe covers a highly conserved region of the *v2r* gene and a MSA (Multiple Sequence Alignment) search in the *X. tropicalis* V2R repertoire finds 75 family members with sequence identity 94 % and above (data not shown). This probe labels a dense population of cells in the VNO (Fig. 6l), presumably due to extended cross-reactivity. However, expression is nearly absent from the MOE, and only occasionally a labeled cell was observed (data not shown, see also [15], well below the expression frequency observed for single receptor genes of about 1–2 cells/section. Due to this near absence in the MOE, genes detected by this probe are not candidates for mediating odor responses in the MOE. In contrast, all *v1r*, class I and II *or* genes and one *taar* gene examined were absent from the VNO and found exclusively within the MOE (Fig. 6), exhibiting sparse expression patterns characteristic of individual olfactory receptor genes [47] and similar to adult, post-metamorphic expression patterns, which have been reported for some of these genes [16, 27, 29]. For *or* and *taar* genes, an expression in the MOE is expected, as both fish and mammals show this pattern. The MOE-specific V1R expression parallels the situation in teleost fish, but not in mammals, which express *v1r* genes generally in the VNO, with few exceptions [48–50]. Thus, the amphibian VNO appears to represent a transition state, morphologically clearly a distinct organ like the mammalian one, but molecularly in an intermediate stage compared to the mammalian VNO.

### Spatial distribution of several *or* genes parallels that of the medial odor processing stream

**Fig. 7:**
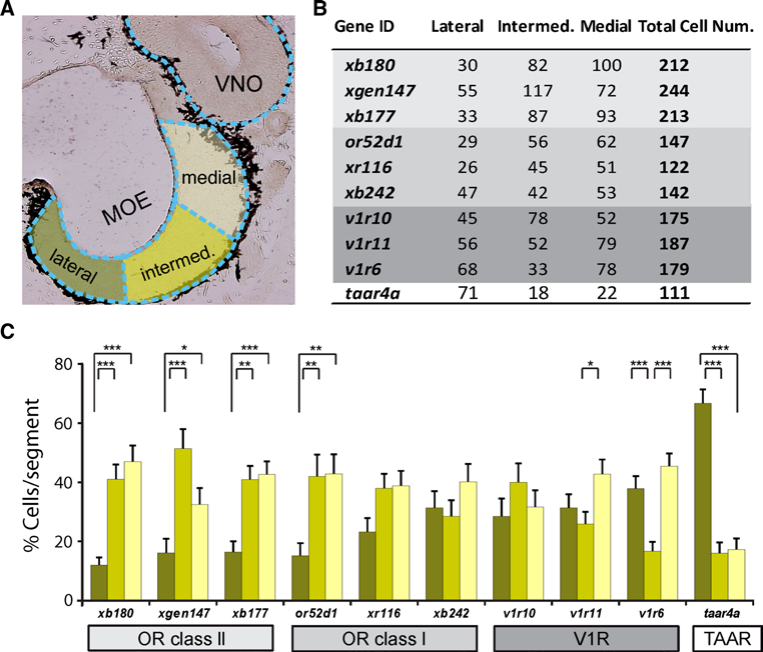
Quantification of lateral-to-medial distribution of ten olfactory receptor genes. **a** Schematic illustration showing the three subdivisions of the MOE, lateral, intermediate, and medial, respectively. **b** Cumulative number of cells counted for each gene in lateral, intermediate, and medial subdivision and total number. *or* class II genes *light grey background*; *or* class I genes, *dark grey background*; *v1r* genes, *middle grey background*; *taar* gene, *white background*. **c** Quantitative evaluation of the spatial distribution for ten genes, same color code as in **a**, lateral (*dark green*), intermediate (*light green*) and medial (*yellow*) parts of the MOE. Percentage of cells in each segment was determined for each section, averaged over sections and shown as mean ± SEM. Significance was determined by *t* test and is denoted by *asterisks*: *p < 0.05, **p < 0.01, ***p < 0.002, *error bars* show SEM

To compare the spatial expression patterns of olfactory receptor genes in the MOE with the previously identified lateral and medial odor processing streams, we quantified the expression frequency for ten different genes by in situ hybridization, applying the previously used subdivision into medial, intermediate, and lateral regions (Fig. 7). About 100 tissue sections were evaluated per gene, resulting in counts of over 100–200 labeled cells per gene. These experiments showed clear and highly significant differences in the spatial distribution of individual olfactory receptor genes.

Notable was a group of genes showing strong and significant depletion in the lateral third of the MOE (*p* < 0.01, Fig. 7). This group encompasses all three class II *or* genes examined and one of the class I *or* genes. Another class I *or* gene shows the same tendency, which, however, does not reach significance. This expression pattern is very similar to the spatial patterns observed for the medial odor processing stream described above, consistent with class II and at least some class I ORs underlying the odor responses associated with the medial stream.

At least one class I *or* and one *v1r* gene are distributed rather evenly between the three subdivisions of the MOE. Such expression patterns could be part of either the lateral or the medial stream, due to the broad and partly overlapping nature of both streams, and therefore no tentative assignment to either of these streams is possible. Interestingly, another *v1r* gene, *v1r6*, shows a distribution not encountered for any of the odor responses and G proteins examined, namely a pronounced depletion in the intermediate region, highly significant in comparison to both the lateral and the medial region. The third *v1r* gene, *v1r11*, exhibits a similar pattern, which however, only reaches significance in the intermediate-to-medial comparison.

### The spatial distribution of the *taar* gene parallels that of the lateral odor processing stream

Somewhat unexpectedly, the *taar* gene investigated showed a very pronounced lateral enrichment (Fig. 7), very similar to the spatial distribution of amino acid responses (Fig. 3). Thus, the *taar* gene might be a candidate for mediating amino acid responses. However, the *taar* gene family in *Xenopus* comprises just three genes [46], one of which is not expressed in the olfactory system (Fig. 6j), which is much less than expected judging from the frequency and diversity of reported amino acid responses [28].

Since a fish V2R has been shown to bind amino acids [51, 52], this large family appeared to be a good candidate for additional receptors with a lateral enrichment.

However, the V2R subclade examined here using a broadly cross-reactive probe very rarely shows any expression in the MOE (data not shown). Furthermore, expression patterns for several genes from other V2R subclades also do not parallel the amino acid response pattern (A.S., S.I.K., unpublished observation).

**Fig. 8:**
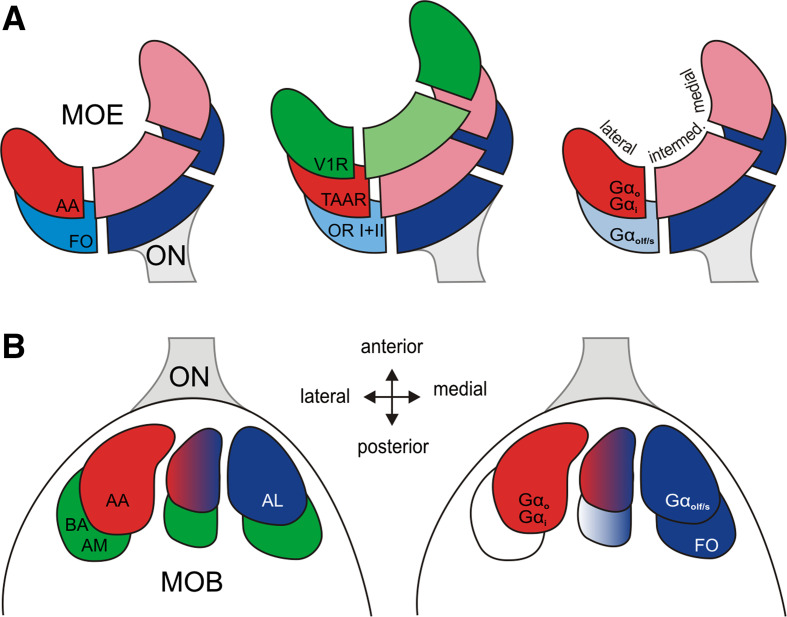
Schematic representation of the lateral and medial olfactory stream in larval *Xenopus laevis*. **a** Spatial distributions observed in the MOE. The lateral stream (*red hues*) is characterized by amino acid responses (*left panel*), *taar* receptors (*middle panel*) and Gα_i_/Gα_o_ (*right panel*). The medial stream (*blue hues*) is represented by forskolin responses, all *or* class II and some *or* class I receptors, and expression of Gα_olf/s_. Some receptors (*green hues*) are homogenously distributed or show a depletion in the intermediate region (*v1r* genes). **b** Spatial distributions observed in the MOB. The lateral stream (*red hues*) is characterized by amino acid responses (*left panel*), and expression of Gα_i_/Gα_o_ (*right panel*). The medial stream (*blue hues*) is represented by responses to alcohols, ketones, aldehydes and forskolin, and expression of Gα_olf/s_. Responses to other odors (bile acids, amines, *green hues*) are rather homogenously distributed. *ON* olfactory nerve, *V1R* vomeronasal receptor genes of type 1, *V2R* vomeronasal receptor genes of type 2, *OR I* odorant receptor genes class I, *OR II* odorant receptor genes class II, *TAAR* trace amine-associated receptors, *AA* mixture of amino acids, *AL* mixture of alcohols, ketones and aldehydes, *AM* mixture of amines, *BA* mixture of bile acids, *FO* forskolin

Taken together, the medial olfactory stream is characterized by expression of class II *or* and at least some class I *or* genes in ciliated ORNs that are activated by, among others, alcohols, aldehydes and ketones and signal via Gα_olf/s_ (Fig. 8, blue hues). The lateral olfactory stream responds, among others, to amino acids, and signals via Gα_i_ and Gα_o_ (Fig. 8, red hues). Its olfactory receptors could include *taar* genes. Interestingly, neuronal responses to bile acids and amines may be carried by both streams (Fig. 8, green hues).

## Discussion

The building principles structuring the olfactory sense of teleost fishes are very different from that of terrestrial mammals. Spatial segregation into different olfactory subsystems and distinct segregation even within such subsystems are a hallmark of mammals [2], whereas a single sensory surface with little segregation is observed in the olfactory system of teleost fishes [53]. It is not known how this major reorganization of the olfactory system took place during evolution of terrestrial vertebrates. The presence of an accessory olfactory system in lungfish [5, 6], a close relative of tetrapods, might indicate that this structure arose early in the evolution of lobe-finned fishes. However, the vomeronasal primordia of lungfish are morphologically very different from the VNO of mammals and may well have arisen as lineage-specific specializations. The pronounced topography of the projection from MOE to olfactory bulb observed in some cartilaginous fish [54] appears to be an example of such lineage-specific specialization, since neither mammals nor teleost fish exhibit this feature.

Here we have examined the olfactory system of the amphibian *X. laevis*, an early diverging tetrapod. We focused this analysis on the larval olfactory system, since adults have undergone complex restructuring during metamorphosis, making them less suitable for comparison to the mammalian (and teleost) olfactory system. Furthermore, *Xenopus* larvae are fully aquatic, allowing the comparison of aquatic with terrestrial tetrapods. Their olfactory epithelium shows clear morphological separation into two subsystems, the MOE and the VNO [42, 55], but the MOE still appears to be much more heterogeneous than the MOE of mammals [15, 27]. We have quantitatively analyzed several molecular and functional parameters within the MOE and olfactory bulb of *X. laevis* to establish the degree of segregation in this olfactory subsystem.

### Incomplete segregation of the vomeronasal system from the main sensory system

Most amphibian species, including *Xenopus*, have both a MOE and a VNO [42, 56]. We have used a broadly crossreactive probe hybridizing with at least 75 different *v2r* genes and find nearly exclusive expression in the VNO. The occasional labeled cells in the MOE most likely represent fuzzy targeting of these *v2r* genes since the frequency of MOE expression is well below that of other individual receptor genes. The expression pattern we observed is similar to the one observed for some *v2r* genes in larval and adult *X. laevis* [15]. We estimate that our probe samples roughly one-fifth of the *Xenopus* V2R repertoire [57]. Several other *v2r* genes we have examined show VNO-specific expression as well (A.S. and S.I.K., unpublished observation). In contrast, we found the *v1r*-like receptor genes to be expressed exclusively in the MOE, like their teleost counterparts (*ora* genes, [58]), and unlike the mammalian *v1r* genes, which are mostly restricted to the VNO ([2], for exceptions see [48, 49]). This expression is retained during the complex morphological reorganization taking place during metamorphosis (cf. [27]).

Taken together, the vomeronasal system appears to have originated as a segregated system for V2R-expressing neurons only. This opens the fascinating possibility that some time after the divergence of amphibians from the tetrapod lineage V1R-expressing neurons may have been reprogrammed for targeting the VNO.

### G proteins in the MOB define the medial and lateral streams

Anatomically, three large and one small ventromedial glomerular cluster can be distinguished in the larval *X. laevis* olfactory bulb [18, 59]. Such glomerular clusters also have been described in other lower vertebrates (see [12, 60–62]). We have shown that the lateral and medial cluster are distinguished by their exclusive and complementary expression of G proteins, non-overlapping afferent pattern at extreme lateral and medial positions, as well as exclusive and complementary responses to two odor groups, amino acids and alcohols, aldehydes and ketones. The intermediate cluster appears to be a transition zone, with mixed identity. These results confirm and extend earlier results by some of the authors [18].

The sharp segregation of Gα_olf/s_ and Gα_i_/Gα_o_ protein expression into a medial and a lateral half of the MOB shows that these G proteins demarcate the medial and the lateral olfactory stream, respectively. The co-expression of Gα_olf/s_ with tubulin, a marker of ciliated neurons, shows the medial stream to be composed of ciliated ORNs. Correspondingly, the co-expression of Gα_i_ and Gα_o_ with phalloidin, a marker for f-actin, shows microvillous ORNs as a main component of the lateral odor processing stream. This is reminiscent of similar G protein spatial patterns in the lamprey and goldfish olfactory system [62, 63], although the segregation is less strict for the fish olfactory system.

### Odor responses to amino acids and alcohols, aldehydes, and ketones are strictly segregated to the lateral and the medial stream, respectively

The complete absence of amino acid-responding glomeruli in the medial olfactory bulb, and the complementary total absence of alcohol, aldehyde, and ketone-responding glomeruli in the lateral olfactory bulb show that there are clear-cut functional differences between the medial and lateral olfactory processing stream. It is worth pointing out that this is not valid for all odor groups, as amines and bile acids are represented in both streams and indeed we find both forskolin-sensitive (cAMP-dependent) and forskolin-insensitive neurons in these two odor groups. Nevertheless, we can conclude that responses to amino acids in *X. laevis* are carried exclusively by microvillous receptor neurons and that responses to alcohols, ketones, and aldehydes are carried exclusively by ciliated receptor neurons. For amino acids, this corresponds to the situation in fishes ([12, 41] but see [11, 32, 33, 64, 65]) and for alcohols, aldehydes, and ketones it is very reminiscent of the mammalian situation [66]. The distribution of amine and bile acid responses to both streams appears to be less specific than in fish (catfish: [11]; zebrafish: [33]; goldfish: [32], but see salmon: [67]), but has some parallels in the mammalian system, where, e.g., amines have been found to elicit responses both in the main and the accessory olfactory system [68].

### The lateral and medial olfactory streams are partially segregated already in the sensory surface

We observe Gα_olf/s_, the marker of the medial stream to be strongly depleted, but not absent in the lateral region of the MOE, and vice versa we find Gα_i_ and Gα_o_, markers of the lateral stream, to be depleted in the medial region of the MOE. The response to amino acids is strongly enriched in the lateral region, similar to the distribution of Gα_i_ and Gα_o_ immunoreactivity, as expected from the co-localization of these parameters in the lateral olfactory bulb. While alcohol, aldehyde, and ketone-responding cells were too sparse to allow an accurate determination of spatial distribution, we find the expected medial enrichment of forskolin responses, which serve as indicator of cAMP-responsive neurons, i.e., ciliated neurons. Interestingly, the forskolin distribution is considerably broader than the Gα_olf/s_ distribution. While we cannot exclude methodological explanations (different thresholds could explain different steepness of the distribution), it is also conceivable that some ciliated neurons may not signal through Gα_olf/s_, as has been observed for lamprey [62].

Medial and lateral enrichment factors range between 1.5 and 3-fold, and are thus clearly less pronounced than the near complete separation we observe for the corresponding parameters in the olfactory bulb. In other words, an incomplete segregation of different functionalities in the olfactory sensory surface is completed en route to the olfactory bulb by further sorting out of the axons of the corresponding receptor neuron subpopulations.

### The medial olfactory stream may be signaling via class II odorant receptors

The distributions so far discussed presumably result from a summation over a multitude of different olfactory receptors, and expression patterns of individual receptor genes might deviate to some extent from the averaged distributions. However, a receptor distribution mimicking those of G proteins or odor responses would allow the hypothesis that such a receptor is expressed in the respective olfactory stream.

We found all three class II and one class I odorant receptor examined to be strongly depleted in the lateral region of the MOE, with ratios very similar to that of Gα_olf/s_. We conclude that some class II (the so-called mammalian-like) *or* genes appear to be expressed in ciliated neurons of the medial olfactory stream. Further analysis will be required to see to what extent this is generalizable to all class II *or* genes. Class I (so-called fish-like) *or* genes may be more heterogeneous as we find already two different spatial patterns (medial enriched and ubiquitous) in the three genes analyzed. No candidates for laterally enriched *or* genes have been found, but we cannot exclude that other class I or class II genes among the large family of *or* genes might show such a distribution.

### A *taar* gene as candidate for the lateral odor-processing stream

Our data do suggest *taar4a* as a receptor gene candidate for the lateral olfactory stream. This came unexpectedly, since ligands for mammalian *taar* genes are believed to be mainly amines [45], which exhibit major biochemical differences to amino acids. In this context, it will be interesting to search for ligands of the *Xenopus* *taar* genes. Furthermore, responses to amino acids are much more frequent than that to the other three odor groups examined, and also very diverse [28], arguing for a sizable group of receptors dedicated to their detection, and not just two genes (of the three *taar* genes present in *Xenopus*, *taar1* is not expressed in the olfactory system).

The *v2r* gene family would appear to contain plausible candidates, since it contains over 300 genes in *Xenopus* [57], and one *v2r* receptor has been shown to be an amino acid receptor in two fish species [51, 52]. However, our probe, crossreacting with at least 75 different V2Rs and representing the largest subclade of the *Xenopus* V2R family [57], shows nearly exclusive expression in the VNO, consistent with findings by Hagino-Yamagishi and coworkers [15]. The occasional labeled cells in the MOE observed by us as well as others [15] are too rare to explain a major reactivity of this tissue. Furthermore, expression patterns for several genes from other V2R subclades also do not parallel the amino acid response pattern (A.S., S.I.K., unpublished observation). However, a truly comprehensive analysis of olfactory receptor families each comprising several hundred genes (*Xenopus* *or*, *v2r* genes, see [57, 69, 70]) is not feasible, and so the molecular nature of at least the majority of amino acid receptors remains an open question for now.

The fourth olfactory receptor gene family, *v1r* genes appear to be unlikely candidates for generating the complete lateral stream, as this rather small population of about two dozen receptors [57, 58] does not appear large enough. Moreover, the three representatives examined here show no lateral enrichment, but in two cases a near homogeneous distribution, and in one case a new pattern, a depletion restricted to the intermediate segment.

### *Xenopus laevis* shows absence of correlation between aquatic life style and spatial segregation tendency

The present study revealed the existence of two distinct olfactory subsystems within the main olfactory system of larval *X. laevis*. A lateral and a medial odor-processing stream show clearly diverging odorant sensitivities, distinct transduction mechanisms with differing G proteins, receptor neurons with different morphology (microvillous vs. ciliated), as well as differences in the expression frequencies of olfactory receptor genes, notwithstanding the absence of lateralization for some odor responses and receptors. These lateral and medial processing streams should not be confused with the segregation of the adult olfactory epithelium into a lateral and a medial diverticulum, which express class I and class II olfactory receptors, respectively [29]. During metamorphosis, complex reorganizations take place, and while the medial enrichment of larval *or* class II receptors is reminiscent of their expression in the medial diverticulum (also named principal cavity, or air nose) in the adult, the ubiquitous or medially enriched distribution of larval *or* class I receptors is clearly different from their restricted expression in the adult lateral diverticulum (other names are middle cavity, water nose). The presence of a vomeronasal epithelium in the adult medial diverticulum (posterolateral area of the principal cavity; see [71]) also has no parallel in the larval olfactory system [15].

Since the transition from aqueous to airborne olfaction has not yet occurred for the fully aquatic tadpoles of *X. laevis*, the distinct, if limited, tendency to spatial segregation appears unrelated to this transition and indeed may be an inherent characteristic of evolution in the lobe-finned fish lineage, cf. [5, 6]. In the ray-finned lineage there exist very few data concerning spatial segregation in the epithelium, but expression of *or* genes shows radially symmetric domains [53] unlike the left/right asymmetry observed here. Backtracing from small regions of the zebrafish olfactory bulb results in widespread labeled cells in the olfactory epithelium [72], unlike the more restricted distributions of backtraced neurons observed here. In conclusion, the olfactory system of *X. laevis* shows distinct, but incomplete segregation in the main olfactory system and thus appears well suited to investigate the molecular driving forces behind such olfactory regionalization.

## Abbreviations

MOE: Main olfactory epithelium
VNO: Vomeronasal organ
ORs: Odorant receptors
V1Rs: Vomeronasal receptors of type 1
V2Rs: Vomeronasal receptors of type 2
AOB: Accessory olfactory bulb
MOB: Main olfactory bulb
TAARs: Trace amine-associated receptors
ORNs: Olfactory receptor neurons

## References

[1] Mombaerts P (2004). Genes and ligands for odorant, vomeronasal and taste receptors. Nat Rev Neurosci.

[2] Munger SD, Leinders-Zufall T, Zufall F (2009). Subsystem organization of the mammalian sense of smell. Annu Rev Physiol.

[3] Mori K, Sakano H (2011). How is the olfactory map formed and interpreted in the mammalian brain?. Annu Rev Neurosci.

[4] Grüneberg H (1973). A ganglion probably belonging to the N. terminalis system in the nasal mucosa of the mouse. Z Anat Entwicklungsgesch.

[5] Gonzalez A, Morona R, Lopez JM, Moreno N, Northcutt RG (2010) Lungfishes, like tetrapods, possess a vomeronasal system. Front Neuroanat 4. Art no 130 10.3389/fnana.2010.00130 PMC2951178 20941371

[6] Nakamuta S, Nakamuta N, Taniguchi K, Taniguchi K (2012). Histological and ultrastructural characteristics of the primordial vomeronasal organ in lungfish. Anat Rec (Hoboken).

[7] Taniguchi K, Saito S, Taniguchi K (2011). Phylogenic outline of the olfactory system in vertebrates. J Vet Med Sci.

[8] Hamdani el H, Doving KB (2007). The functional organization of the fish olfactory system. Prog Neurobiol.

[9] Korsching S (2009). The molecular evolution of teleost olfactory receptor gene families. Results Probl Cell Differ.

[10] Oka Y, Korsching SI (2011). Shared and unique G alpha proteins in the zebrafish versus mammalian senses of taste and smell. Chem Senses.

[11] Hansen A, Rolen SH, Anderson K, Morita Y, Caprio J (2003). Correlation between olfactory receptor cell type and function in the channel catfish. J Neurosci.

[12] Sato Y, Miyasaka N, Yoshihara Y (2005). Mutually exclusive glomerular innervation by two distinct types of olfactory sensory neurons revealed in transgenic zebrafish. J Neurosci.

[13] Hayden S, Bekaert M, Crider TA, Mariani S, Murphy WJ (2010). Ecological adaptation determines functional mammalian olfactory subgenomes. Genome Res.

[14] Manzini I, Schild D (2010) Olfactory Coding in Larvae of the African Clawed Frog *Xenopus laevis*. In: Menini A (ed) The Neurobiology of Olfaction. Chap 4, CRC Press, Boca Raton 21882433

[15] Hagino-Yamagishi K, Moriya K, Kubo H, Wakabayashi Y, Isobe N (2004). Expression of vomeronasal receptor genes in *Xenopus laevis*. J Comp Neurol.

[16] Mezler M, Konzelmann S, Freitag J, Rössler P, Breer H (1999). Expression of olfactory receptors during development in *Xenopus laevis*. J Exp Biol.

[17] Ji Y, Zhang Z, Hu Y (2009). The repertoire of G-protein-coupled receptors in *Xenopus tropicalis*. BMC Genomics.

[18] Manzini I, Heermann S, Czesnik D, Brase C, Schild D (2007). Presynaptic protein distribution and odour mapping in glomeruli of the olfactory bulb of *Xenopus laevis* tadpoles. Eur J Neurosci.

[19] Nieuwkoop PD, Faber J (1994). Normal table of *Xenopus laevis* (Daudin).

[20] Czesnik D, Rössler W, Kirchner F, Gennerich A, Schild D (2003). Neuronal representation of odourants in the olfactory bulb of *Xenopus laevis* tadpoles. Eur J Neurosci.

[21] Manzini I, Schild D (2003). cAMP-independent olfactory transduction of amino acids in *Xenopus laevis* tadpoles. J Physiol.

[22] Manzini I, Schild D (2003). Multidrug resistance transporters in the olfactory receptor neurons of *Xenopus laevis* tadpoles. J Physiol.

[23] Manzini I, Schweer TS, Schild D (2008). Improved fluorescent (calcium indicator) dye uptake in brain slices by blocking multidrug resistance transporters. J Neurosci Methods.

[24] Junek S, Chen TW, Alevra M, Schild D (2009). Activity correlation imaging: visualizing function and structure of neuronal populations. Biophys J.

[25] Hassenklöver T, Kurtanska S, Bartoszek I, Junek S, Schild D (2008). Nucleotide-induced Ca2+ signaling in sustentacular supporting cells of the olfactory epithelium. Glia.

[26] Mezler M, Fleischer J, Conzelmann S, Korchi A, Widmayer P (2001). Identification of a nonmammalian Golf subtype: functional role in olfactory signaling of airborne odorants in *Xenopus laevis*. J Comp Neurol.

[27] Date-Ito A, Ohara H, Ichikawa M, Mori Y, Hagino-Yamagishi K (2008). *Xenopus* V1R vomeronasal receptor family is expressed in the main olfactory system. Chem Senses.

[28] Manzini I, Schild D (2004). Classes and narrowing selectivity of olfactory receptor neurons of *Xenopus laevis* tadpoles. J Gen Physiol.

[29] Freitag J, Krieger J, Strotmann J, Breer H (1995). Two classes of olfactory receptors in *Xenopus laevis*. Neuron.

[30] Gliem S, Schild D, Manzini I (2009). Highly specific responses to amine odorants of individual olfactory receptor neurons in situ. Eur J Neurosci.

[31] Carr WES, Derby CD (1986). Chemically stimulated feeding-behavior in marine animals—importance of chemical-mixtures and involvement of mixture interactions. J Chem Ecol.

[32] Rolen SH, Sorensen PW, Mattson D, Caprio J (2003). Polyamines as olfactory stimuli in the goldfish *Carassius auratus*. J Exp Biol.

[33] Michel WC, Sanderson MJ, Olson JK, Lipschitz DL (2003). Evidence of a novel transduction pathway mediating detection of polyamines by the zebrafish olfactory system. J Exp Biol.

[34] Sorensen PW, Caprio J, Evans DH (1998). Chemoreception. The physiology of fishes.

[35] Altner H (1962). Untersuchungen über Leistungen und Bau der Nase des südafrikanischen Krallenfrosches *Xenopus laevis* (Daudin, 1803). Z Vlg Physiol.

[36] Manzini I, Brase C, Chen TW, Schild D (2007). Response profiles to amino acid odorants of olfactory glomeruli in larval *Xenopus laevis*. J Physiol.

[37] Touhara K (2002). Odor discrimination by G protein-coupled olfactory receptors. Microsc Res Tech.

[38] Manzini I, Rössler W, Schild D (2002). cAMP-independent responses of olfactory neurons in *Xenopus laevis* tadpoles and their projection onto olfactory bulb neurons. J Physiol.

[39] Friedrich RW, Korsching SI (1998). Chemotopic, combinatorial, and noncombinatorial odorant representations in the olfactory bulb revealed using a voltage-sensitive axon tracer. J Neurosci.

[40] Korsching SI (2001). Odor maps in the brain: spatial aspects of odor representation in sensory surface and olfactory bulb. Cell Mol Life Sci.

[41] Friedrich RW, Korsching SI (1997). Combinatorial and chemotopic odorant coding in the zebrafish olfactory bulb visualized by optical imaging. Neuron.

[42] Hansen A, Reiss JO, Gentry CL, Burd GD (1998). Ultrastructure of the olfactory organ in the clawed frog, *Xenopus laevis*, during larval development and metamorphosis. J Comp Neurol.

[43] Wieland T (1987). 50 years of phalloidine: its discovery, characterization and current and future applications in cell research. Naturwissenschaften.

[44] Corbit KC, Aanstad P, Singla V, Norman AR, Stainier DY (2005). Vertebrate smoothened functions at the primary cilium. Nature.

[45] Liberles SD, Buck LB (2006). A second class of chemosensory receptors in the olfactory epithelium. Nature.

[46] Hussain A, Saraiva LR, Korsching SI (2009). Positive Darwinian selection and the birth of an olfactory receptor clade in teleosts. Proc Natl Acad Sci USA.

[47] Strotmann J, Wanner I, Helfrich T, Beck A, Meinken C (1994). Olfactory neurones expressing distinct odorant receptor subtypes are spatially segregated in the nasal neuroepithelium. Cell Tissue Res.

[48] Karunadasa DK, Chapman C, Bicknell RJ (2006). Expression of pheromone receptor gene families during olfactory development in the mouse: expression of a V1 receptor in the main olfactory epithelium. Eur J Neurosci.

[49] Wakabayashi Y, Mori Y, Ichikawa M, Yazaki K, Hagino-Yamagishi K (2002). A putative pheromone receptor gene is expressed in two distinct olfactory organs in goats. Chem Senses.

[50] Rodriguez I, Greer CA, Mok MY, Mombaerts P (2000). A putative pheromone receptor gene expressed in human olfactory mucosa. Nat Genet.

[51] Speca DJ, Lin DM, Sorensen PW, Isacoff EY, Ngai J (1999). Functional identification of a goldfish odorant receptor. Neuron.

[52] Luu P, Acher F, Bertrand HO, Fan J, Ngai J (2004). Molecular determinants of ligand selectivity in a vertebrate odorant receptor. J Neurosci.

[53] Weth F, Nadler W, Korsching S (1996). Nested expression domains for odorant receptors in zebrafish olfactory epithelium. Proc Natl Acad Sci USA.

[54] Meredith T, Hansen A (2010). Hemi-bulb organization in the elasmobrach brain. Chem Senses.

[55] Oikawa T, Suzuki K, Saito TR, Takahashi KW, Taniguchi K (1998). Fine structure of three types of olfactory organs in *Xenopus laevis*. Anat Rec.

[56] Taniguchi K, Saito S, Oikawa T, Taniguchi K (2008). Phylogenic aspects of the amphibian dual olfactory system. J Vet Med Sci.

[57] Shi P, Zhang J (2007). Comparative genomic analysis identifies an evolutionary shift of vomeronasal receptor gene repertoires in the vertebrate transition from water to land. Genome Res.

[58] Saraiva LR, Korsching SI (2007). A novel olfactory receptor gene family in teleost fish. Genome Res.

[59] Gaudin A, Gascuel J (2005). 3D atlas describing the ontogenic evolution of the primary olfactory projections in the olfactory bulb of *Xenopus laevis*. J Comp Neurol.

[60] Riddle DR, Wong LD, Oakley B (1993). Lectin identification of olfactory receptor neuron subclasses with segregated central projections. J Neurosci.

[61] Baier H, Korsching S (1994). Olfactory glomeruli in the zebrafish form an invariant pattern and are identifiable across animals. J Neurosci.

[62] Frontini A, Zaidi AU, Hua H, Wolak TP, Greer CA (2003). Glomerular territories in the olfactory bulb from the larval stage of the sea lamprey *Petromyzon marinus*. J Comp Neurol.

[63] Hansen A, Anderson KT, Finger TE (2004). Differential distribution of olfactory receptor neurons in goldfish: structural and molecular correlates. J Comp Neurol.

[64] Lo YH, Bradley TM, Rhoads DE (1993). Stimulation of Ca(2+)-regulated olfactory phospholipase C by amino acids. Biochemistry.

[65] Ma L, Michel WC (1998). Drugs affecting phospholipase C-mediated signal transduction block the olfactory cyclic nucleotide-gated current of adult zebrafish. J Neurophysiol.

[66] Johnson BA, Leon M (2007). Chemotopic odorant coding in a mammalian olfactory system. J Comp Neurol.

[67] Lo YH, Bellis SL, Cheng LJ, Pang J, Bradley TM (1994). Signal transduction for taurocholic acid in the olfactory system of Atlantic salmon. Chem Senses.

[68] Tirindelli R, Dibattista M, Pifferi S, Menini A (2009). From pheromones to behavior. Physiol Rev.

[69] Niimura Y, Nei M (2005). Evolutionary dynamics of olfactory receptor genes in fishes and tetrapods. Proc Natl Acad Sci USA.

[70] Shi P, Zhang J (2009). Extraordinary diversity of chemosensory receptor gene repertoires among vertebrates. Results Probl Cell Differ.

[71] Föske H (1934). Das Geruchsorgan von *Xenopus laevis*. Z Anat Entwicklungsgesch.

[72] Baier H, Rotter S, Korsching S (1994). Connectional topography in the zebrafish olfactory system: random positions but regular spacing of sensory neurons projecting to an individual glomerulus. Proc Natl Acad Sci USA.

